# Microbial communities and functions changed in rhizosphere soil of *Pinus massoniana* provenances with different carbon storage

**DOI:** 10.3389/fmicb.2023.1264670

**Published:** 2023-11-03

**Authors:** Zichen Huang, Xin He, Chi Zhang, Mengyang Zhang, Jiannan Wang, Yanqing Hou, Dengbao Wang, Sheng Yao, Qiong Yu, Kongshu Ji

**Affiliations:** ^1^State Key Laboratory of Tree Genetics and Breeding, Co-Innovation Center for Sustainable Forestry in Southern China, Nanjing Forestry University, Nanjing, China; ^2^Co-Innovation Center for Sustainable Forestry in Southern China, College of Biology and the Environment, Nanjing Forestry University, Nanjing, China

**Keywords:** *Pinus massoniana*, provenance, carbon storage, microbiome, structural responses

## Abstract

**Introduction:**

The average carbon storage of *Pinus massoniana* is much higher than the average carbon storage of Chinese forests, an important carbon sink tree species in subtropical regions of China. However, there are few studies on the differences in rhizosphere microorganisms of *P. massoniana* with different carbon storages.

**Methods:**

To clarify the relationships between plant carbon storage level, environmental parameters and microbial community structure, we identified three carbon storage levels from different *P. massoniana* provenances and collected rhizosphere soil samples. We determined chemical properties of soil, extracellular enzyme activity, and microbial community structures at different carbon storage levels and examined how soil factors affect rhizosphere microorganisms under different carbon storage levels.

**Results:**

The results revealed that soil organic carbon (SOC), nitrate nitrogen (NO_3_^−^-N), ammonium nitrogen (NH_4_^+^-N) contents all increased with increasing carbon storage levels, while pH decreased accordingly. In contrast, the available phosphorus (AP) content did not change significantly. The soil AP content was within the range of 0.91 ~ 1.04 mg/kg. The microbial community structure of *P. massoniana* changed with different carbon storage, with *Acidobacteria* (44.27%), *Proteobacteria* (32.57%), and *Actinobacteria* (13.43%) being the dominant bacterial phyla and *Basidiomycota* (73.36%) and *Ascomycota* (24.64%) being the dominant fungal phyla across the three carbon storage levels. Soil fungi were more responsive to carbon storage than bacteria in *P. massoniana*. C/N, NH_4_^+^-N, NO_3_^−^-N, and SOC were the main drivers (*p* < 0.05) of changes in rhizosphere microbial communities.

**Discussion:**

The results revealed that in the rhizosphere there were significant differences in soil carbon cycle and microorganism nutrient preferences at different carbon storages of *P. massoniana* provenance, which were significantly related to the changes in rhizosphere microbial community structure. Jiangxi Anyuan (AY) provenance is more suitable for the construction of high carbon storage plantation.

## 1. Introduction

Excessive carbon emissions exacerbate climate warming and pose serious challenges to human society and the earth’s ecosystems (Liu and Deng, 2011). Forests are an important part of the land ecosystem and contribute most of the aboveground carbon and nearly half of the underground carbon, playing a crucial part in regulating the global carbon cycle as well as coping with climate change (Yun et al., 2018). Approximately 77% of the global terrestrial carbon pool is stored in forest ecosystems (Wang and Wang, 2015). Estimation of forest ecosystem carbon storage will help us better understand the role of forests in global warming (Fang and Wang, 2001). The accumulation method is a common method to estimate forest carbon storage. The average capacity (t/m) of main tree species in forest was calculated by sampling and measuring. The biomass was calculated according to the total forest stock, and the carbon storage amount was calculated according to the conversion coefficient between biomass and carbon amount (Williams et al., 2000). Forest carbon storage are closely related to environmental factors. Research (Hagedorn et al., 2003; Yue et al., 2018) has shown that soil C/N and total nitrogen content are significantly correlated with carbon storage of forest ecosystems. However, there are few systematic studies on the correlation between the forests carbon storage and rhizosphere soil microorganisms.

For the forest ecosystem, the rhizosphere microecology is an important hub connecting the aboveground and underground parts of the forest. The rhizosphere generally means the microarea of the root axis surface within a few millimeters, including three important components of root-soil-microorganism (Schweinsberg-Mickan et al., 2012). There are highly diverse microbial communities in the rhizosphere. These microorganisms are extremely susceptible to influences from the external environment (Zhang X. et al., 2020). At the same time, the microorganisms can interact with the root system and profoundly affect features, such as plant biomass and yield (Zhang et al., 2021b). Researchers have confirmed that root carbon input can affect the microbial community of rhizosphere (Wen et al., 2022). In turn, plant growth process can be affected by some rhizosphere microorganisms (Chiboub et al., 2020). Therefore, it is necessary to further clarify the correlation between the carbon storage of *Pinus massoniana* and rhizosphere microorganisms.

As the most common and largest forest species in southern China, *P. massoniana* plays a crucial part in wood-produce, carbon storage and other ecological services (You et al., 2018). The natural *P. massoniana* forest has a storage volume of 586.99 million m^3^. The artificial forest has a storage volume of 321.22 million m^3^, the third tree species, second only to *Cunninghamia lanceolata* and poplar (Ji et al., 2022). In Guangxi, China, the carbon storage of artificial arbor trees is 1.07*10^6^ t, and that of *P. massoniana* accounts for 25.1% of the total carbon storage of arbor forests, with a carbon storage of 3.37*10^5^ t (Lan et al., 2019). In addition, in regions such as Jiangxi, Guizhou, and Hunan provinces in China, the total value generated by various ecological service functions of *P. massoniana* is much greater than that of other tree species (Wang and Wang, 2010; Huang X. et al., 2019; Gao et al., 2022). The carbon storage capacity of different *P. massoniana* provenances was significantly different (Zhang Y. et al., 2020). Many researchers have systematically analyzed the correlation between the carbon storage and soil nutrients in *P. massoniana* forests. A positive correlation between the vegetation carbon storage and the content of soil organic carbon in *P. massoniana* forest was revealed (Sun et al., 2020). Nevertheless, there are few systematic studies on the relationship between microorganism of rhizosphere soil and vegetation carbon storage in *P. massoniana* forests. Therefore, it is necessary to continue exploring the relationship between the vegetation carbon storage and the microorganism, environmental factors of rhizosphere soil in *P. massoniana* forests. This will assist with the development of *P. massoniana* forest ecosystem.

In this study, the chemical properties and extracellular enzyme activity of *P. massoniana* provenances rhizosphere soil with varying levels of carbon storage was investigated, as well as the diversity and community structures of bacteria and fungi. Our goal was (1) to evaluate the differences in bacterial and fungal community structure and diversity in *P. massoniana* rhizosphere soil at different levels of carbon storage. (2) To clarify the relationship between plant carbon storage, environmental parameters and microbes. (3) To assess the microbial mechanism of carbon storage differences in different *P. massoniana* provenances, and select the provenances with good interaction relationship with environmental microorganisms for the purpose of building sustainable high carbon storage plantation. Few researchers have directly linked the amount of plant carbon storage of provenances with rhizosphere microorganisms, especially in *P. massoniana*. Therefore, we hypothesize that (1) rhizosphere microbial communities and function of *P. massoniana* provenances will change under different carbon storage levels. (2) Soil environmental factors are the direct causes of rhizosphere microbiome variations. This study combines forest carbon storage with soil microecology, which is helpful for the construction of forest ecosystems with good provenance.

## 2. Materials and methods

### 2.1. Study site and experimental design

The experimental site location is State-owned Forest seed Farm in Yu’an District, Lu′an City, Anhui Province, China (116°12′E, 31°40’N). With an annual rainfall of 1239.8 mm, the climate of this area is warm and humid subtropical monsoon. The frost free period is nearly 225 days, with an annual average temperature of 15°C, the altitude is 80 ~ 110 m, and the slope is 5° ~ 15°. The soil is mostly clayey yellow soil, with a pH of 5.5 ~ 6.5. The *P. massoniana* provenance trial forest was established in the spring of 1981. There are 64 provenances in total with a completely randomized block design. The site is divided into six evenly divided districts (Supplementary Figure S1). Each district group contains 64 plots, i.e., 64 provenances, and each provenance has 9 plants as a plot (3 rows, 3 plants per row). The experimental forest is 41 years old and has not been thinned during the period, with a preservation rate of 36.52%. The number of surviving trees in some provenances cannot meet the requirements of statistical analysis due to the growth competition among individual trees, diseases and insect pests in the late growth stage. Eventually, there are 55 provenances that met the requirement of statistical analysis. We use the binary model in the Chinese forestry industry standard (LY/T2263-2014) to estimate the biomass of various sources (Zeng et al., 2010).


$$M_{A}=0.066615D^{2.09317}H^{0.49763}$$

$$M_{B}=0.008828D^{2.73828}H^{-0.080255}$$

*M_A_*: total biomass of aboveground organs for *P. massoniana*.

*M_B_*: total biomass of the underground part (i.e., root) of *P. massoniana*.

*D*: diameter at breast height (DBH), *H*: height.

We used the proportional function weighting method to control and calculate the biomass of each organ and then summed it. Since the trunk accounts for the largest portion of the whole standing tree, the relative proportion of the trunk biomass to the total biomass was set as 1, and “g” was set as the proportion function for different organs to the total biomass. “M” was set as the biomass of different organs. We obtain the following regression equation:


$$g_{bark}=0.48729H^{-0.60207}$$

$$g_{branch}=1.59113D^{0.96127}H^{-1.80294}$$

$$g_{leaf}=2.98814D^{0.61586}H^{-2.02349}$$

$$M_{trunk}=1/{\left(1+g_{bark}+g_{branch}+g_{leaf}\right)}^{*}M_{A}$$

$$M_{bark}=g_{1}/{\left(1+g_{bark}+g_{branch}+g_{leaf}\right)}^{*}M_{A}$$

$$M_{branch}=g_{2}/{\left(1+g_{bark}+g_{branch}+g_{leaf}\right)}^{*}M_{A}$$

$$M_{leaf}=g_{3}/{\left(1+g_{bark}+g_{branch}+g_{leaf}\right)}^{*}M_{A}$$

We multiplied the biomass of different organs estimated by the model by the corresponding carbon content coefficient (Table 1) (Zeng and Xiao, 2011). The carbon storage of each organ was obtained. The carbon storage of each plant from various sources was obtained by summing. Based on the calculation results, we selected the first, twenty-eighth, and fifty-fifth ranked carbon storage among the 55 provenances representing high, medium and low carbon storage levels, respectively. They are Jiangxi Anyuan (AY), Fujian Shaowu (SW), and Zhejiang Shengxian (SX) namely (Table 2).

**Table 1:** Carbon content coefficient of different tissues of *P. massoniana.*

Trunk	Bark	Branch	Leaf	Root
0.52 ± 0.01	0.49 ± 0.01	0.52 ± 0.05	0.58 ± 0.02	0.51 ± 0.03

**Table 2:** Biomass and carbon storage of the three *P. massoniana* provenances.

Provenance	height/m mean	DBH/cm mean	Biomass/kg mean	Carbon storage/kg mean
AY	18.90	22.00	218.71	109.42
SW	16.30	19.40	156.27	80.99
SX	14.50	16.10	86.06	43.10

DHB, diameter at breast height.

### 2.2. Soil sampling

Four *P. massoniana* with the same DBH and close to the average value were selected from each provenance. Each tree was set with a 0 ~ 20 cm profile in three directions (120°) to collect fine roots with root diameters less than 2 mm. For the rhizosphere soil, a sterile brush was used to collect the soil within 5 mm of the root surface. Soil mixing of three direction profiles was taken as a repeat, with a total of 4 repeats. Soils were divided into three parts for the determination of different indicators. One part was used directly to determine soil enzyme activity, another part was air-dried for the determination of soil physical and chemical properties, and the last part was frozen at −80°C for microbial group analysis.

### 2.3. Soil factor analysis

Using a glass composite electrode to determine pH value of soil with a water and soil ratio of 1:2.5. The moisture content of the soil was measured by drying it followed by cooling it. Determinations of soil organic carbon (SOC) and available phosphorus (AP) were traditional methods, namely, the potassium dichromate external heating method and molybdenum-antimony-scandium colorimetry (Yeomans and Bremner, 2008). A flow analyzer (Bran+Luebbe, GmbH, Germany) (Bunch and Bernot, 2012) was used to determine the total nitrogen (TN), nitrate nitrogen (NO_3_^−^-N) and ammonium nitrogen (NH_4_^+^-N) contents of the soil. Nitrite reductase (NIR) is a key enzyme in the nitrogen cycle that degrades nitrite to NO or NH_3_. Peroxidase (POD) and polyphenol oxidase (PPO) are key enzymes in the carbon cycle and are mainly associated with the degradation of difficult-to-degrade lignin (Yin et al., 2016). The activities of NIR, PPO, and POD were determined by the 96-well plate microplate method (Saiya-Cork et al., 2002).

### 2.4. 16S rRNA and ITS extraction and PCR process

DNA was extracted from soil samples by using a DNA Isolation Kit (Bio Teke, Beijing, China). The primers 343F-798R (5′- TACGGRAGGCAGCAG -3′/5′- AGGGTATCTAATCCT- 3′) and ITS1F-ITS2 (5′- CTTGGTCATTTAGAGGAAGTAA-3′/5’-GCTGCGTTCTTCATCGATGC- 3′) were used to analyze the bacterial 16S rRNA V3-V4 region and the ITS genes of the fungi (Chen et al., 2020). PCR amplification and Illumina NovaSeq sequencing were conducted at Shanghai OE Biotech. Co., Ltd. (Shanghai, China).

### 2.5. Sequence processing and data analysis

The Illumina MiSeq platform was used to generate the original paired-end sequences. The project can be accessed in NCBI under BioProject ID PRJNA1018330. Primers for raw data sequences were cut out by cutadapt. Representative sequences and ASV abundance tables were generated by DADA2 using the Qiime2 (Bolyen et al., 2019) default parameters. Representative sequences and ASV abundance tables were generated. Silva database (Version 138) was used to annotate the species database. We normalized each sample to a uniform sequence number to analyze the alpha diversity and principal coordinate analysis (PCoA). The tags used for 16S analysis are 3,232 and the tags used for ITS analysis are 19,217. We used the ggplot2 package in R (Ginestet, 2011) to perform alpha indices, principal coordinate analysis (PCoA) of Bray-Curtis distances and community structure barplot. Redundancy analysis (RDA) and the Mantel test were conducted by the vegan package in R (Dixon, 2003). The taxa exhibiting the most significant intergroup variation were identified by linear discriminant analysis (LDA) effect size (LEfSe). Bacterial functions are predicted using the FAPROTAX databases.^1^ FUNGuild databases^2^ were used to annotate fungal functions.

## 3. Results

### 3.1. Soil properties

Table 3 shows the soil property variations of the three provenances. The pH of the rhizosphere soil varied from 4.85 to 5.05. The pH of SX provenance soil was significantly higher than that of AY and SW provenances soils (*p* < 0.05). SOC, NO_3_^−^-N, and NH_4_^+^-N contents of the three groups were significantly different (*p* < 0.05), increasing with increasing carbon storage level. We also observed that the TN content of AY was significantly higher than SW. There was no significant change in soil moisture and AP. SW had significantly greater NIR than AY and SX (*p* < 0.05). Conversely, the POD of AY and SX was significantly higher than SW (*p* < 0.05). The PPO activity did not change significantly with increasing carbon storage level.

**Table 3:** Rhizosphere soil properties of *P. massoniana* provenances at different carbon storage levels.

	SX	SW	AY
pH	5.05 ± 0.02a	4.84 ± 0.13b	4.90 ± 0.43b
Moisture	0.13 ± 0.02	0.12 ± 0.04	0.14 ± 0.01
SOC (g/kg)	8.83 ± 0.76c	16.54 ± 0.18b	25.92 ± 1.48a
TN (g/kg)	1.26 ± 0.15ab	1.13 ± 0.25b	1.47 ± 0.0.16a
C/N	7.10 ± 0.88b	15.27 ± 3.46a	17.80 ± 1.69a
AP (mg/kg)	0.91 ± 0.09	1.02 ± 0.06	1.04 ± 0.05
NH_4_^+^-N (μg/g)	5.22 ± 0.82c	7.60 ± 0.02b	8.99 ± 0.37a
NO_3_^−^-N (μg/g)	0.56 ± 0.59c	5.48 ± 1.26b	15.59 ± 0.61a
POD (nmol/h/g)	2619.74 ± 365.83a	1718.96 ± 251.35b	2324.15 ± 255.85a
PPO (nmol/h/g)	283.42 ± 31.72	243.56 ± 31.14	285.45 ± 56.12
NIR (μmol/d/g)	15.21 ± 2.78b	23.80 ± 3.00a	13.01 ± 0.98b

AY, Jiangxi Anyuan provenance; SW, Fujian Shaowu provenance; SX, Zhejiang Shengxian provenance; Different letters indicate significant differences among treatments using one-way ANOVA (LSD, *p* < 0.05).

### 3.2. Community diversity and richness of soil bacteria and fungi

**Figure 1:**
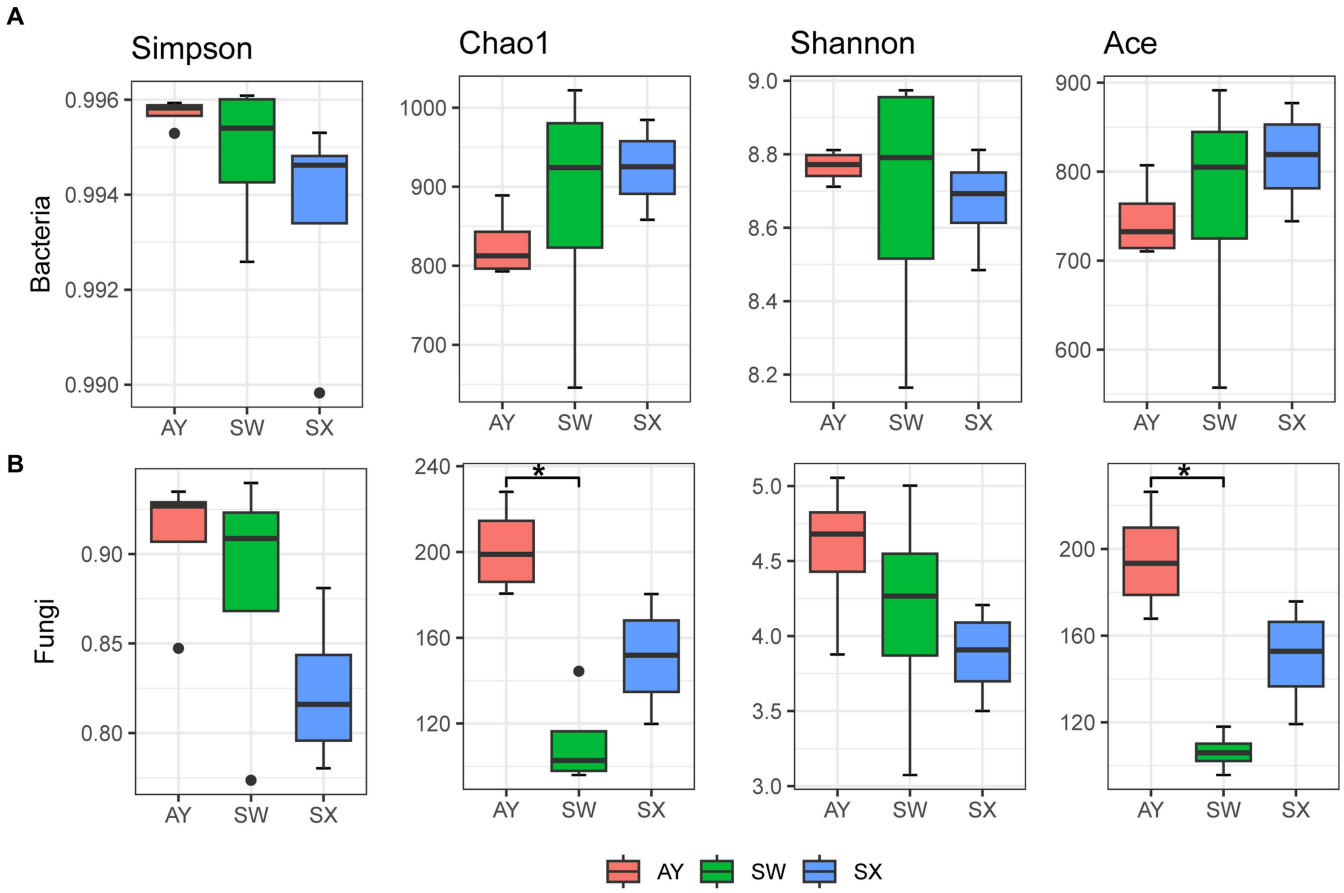
Differences in bacterial **(A)** and fungal **(B)** alpha diversity among the soil of *P. massoniana* provenances at different carbon storage levels. (AY, Jiangxi Anyuan provenance; SW, Fujian Shaowu provenance; SX, Zhejiang Shengxian provenance; “*” indicate that the means of alpha diversity index are significantly different (*p* < 0.05) among different carbon storage levels).

The alpha diversity of bacteria and fungi was estimated by Shannon, Simpson, Chao1, and Ace indices (Figure 1). For bacteria, alpha diversity did not show significant differences among the three provenances. However, the Chao1 and Ace indices generally showed a downward trend with increasing carbon storage level. For fungi, the order for both Chao1 and Ace estimators was AY > SX > SW. AY had significantly greater Chao1 and Ace estimators than SW (*p* < 0.05). The Simpson and Shannon indices of fungi showed an upward trend with increasing carbon storage level.

**Figure 2:**
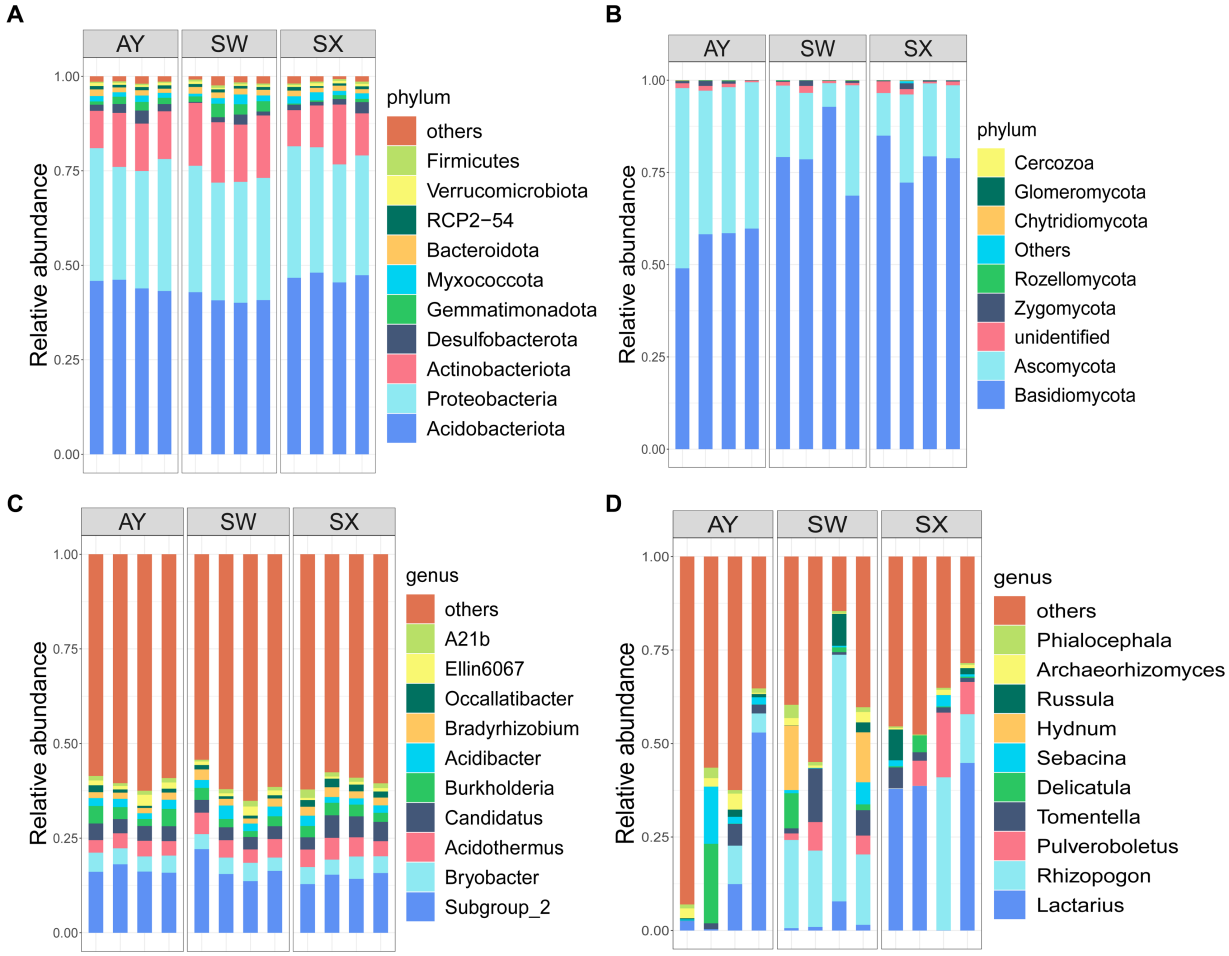
Relative abundance of bacterial **(A,C)** and fungal **(B,D)** phyla and genera among the soil of *P. massoniana* provenances at different carbon storage levels. (AY, Jiangxi Anyuan provenance; SW, Fujian Shaowu provenance; SX, Zhejiang Shengxian provenance. Four bars in the *x*-axis of different groups represent four replicates in each group).

For bacteria, the dominant phyla that we identified were Acidobacteriota (44.27%), Proteobacteria (32.57%), Actinobacteriota (13.43%), Desulfobacterota (1.79%), Gemmatimonadota (1.65%), Myxococcota (1.55%), and Bacteroidota (1.43%). Other species, such as Verrucomicrobiota, Firmicutes and Nitrospirota, accounted for only a small part of identified bacteria (Figure 2A) (Supplementary Table S1). For fungal phyla, the most abundant species were Basidiomycota (73.36%) and Ascomycota (24.64%). Zygomycota, Rozellomycota, Chytridiomycota, and Glomeromycota accounted for only a small part of identified fungi (Figure 2B) (Supplementary Table S1).

At the genus level, the dominant bacteria genus that we identified were *Subgroup_2* (16.01%)*, Bryobacter* (4.48%)*, Acidothermus* (4.44%)*, Candidatus* (4.10%)*, Burkholderia* (2.96%)*, Acidibacter* (2.19%), *Bradyrhizobium* (1.95%)*, Occallatibacter* (1.26%)*, Ellin6067* (1.24%)*, A21b* (1.15%) (Figure 2C). Fungi ASVs mainly comprised *Lactarius* (16.71%), *Rhizopogon* (16.53%), *Pulveroboletus* (3.93%), *Tomentella* (3.59), *Delicatula* (3.26%), *Sebacina* (2.66%), *Hydnum* (2.55%), *Russula* (2.02%), *Archaeorhizomyces* (1.49%) (Figure 2D).

### 3.3. Comparison of bacterial and fungal community structures

**Figure 3:**
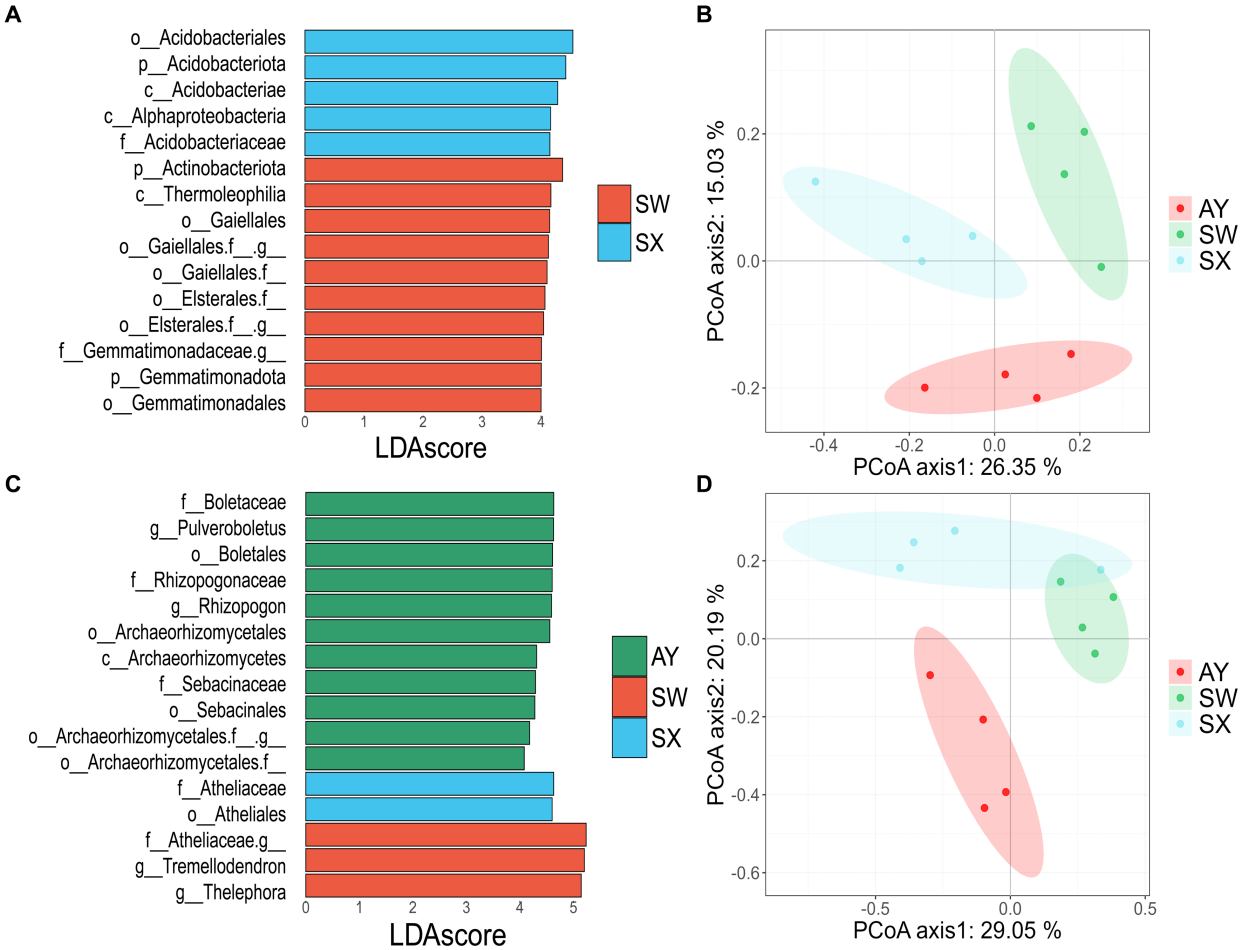
Differences identified using LefSe in bacteria **(A)** and fungi **(B)** of soil of *P. massoniana* provenances at different carbon storage levels. Principal coordinate analysis of Bray-Curtis distances for soil bacterial **(C)** and fungal **(D)** communities in the *P. massoniana* plantation. (AY, Jiangxi Anyuan provenance; SW, Fujian Shaowu provenance; SX, Zhejiang Shengxian provenance).

We performed LEfSe analysis to identify the major biomarkers in *P. massoniana* rhizosphere soil under different carbon storage levels (LDA score > 4) (Figures 3A,B) (Supplementary Table S2). Bacterial taxa mostly included *Acidobacteriota*, *Proteobacteria* and *Actinobacteriota*. Fungi groups were mostly from *Basidiomycota* and *Ascomycota*. SX was enriched in five bacterial taxonomic groups (*Acidobacteriales, Acidobacteriota, Acidobacteriae, Alphaproteobacteria* and *Acidobacteriaceae_Subgroup_1*) and two fungal taxonomic groups (*Boletaceae* and *Pulveroboletus*). The main biomarkers of SW were *Actinobacteriota, Thermoleophilia, Gaiellales, Gemmatimonadota, Gemmatimonadales, Boletales, Rhizopogonaceae, Rhizopogon* and some uncultured families and groups from *Gaiellales, Elsterales*, and *Gemmatimonadaceae*. Taxa with higher abundance in AY were *Archaeorhizomycetales, Archaeorhizomycetes, Sebacinaceae, Sebacinales, Athelicaceae, Atheliales, Tremellodendr, Thelephor*, and some unidentified families from *Archaeorhizomycetales* and *Atheliaceae*.

PCoA based on the Bray-Curtis distance indicated that the community structures of the bacteria and fungi changed at different carbon storage levels (Figures 3C,D). The ANOSIM also supported the result that there were significant differences among the three groups (*p* = 0.001) (Supplementary Figure S2). For bacteria, there were significant differences between AY vs. SW, SW vs. SX, and AY vs. SX (*p* = 0.034, 0.02, 0.04, respectively). For fungi, there were significant differences between AY vs. SW (*p* = 0.026) (Supplementary Figure S2).

### 3.4. Relationships between environmental factors and microbial community structures

**Figure 4:**
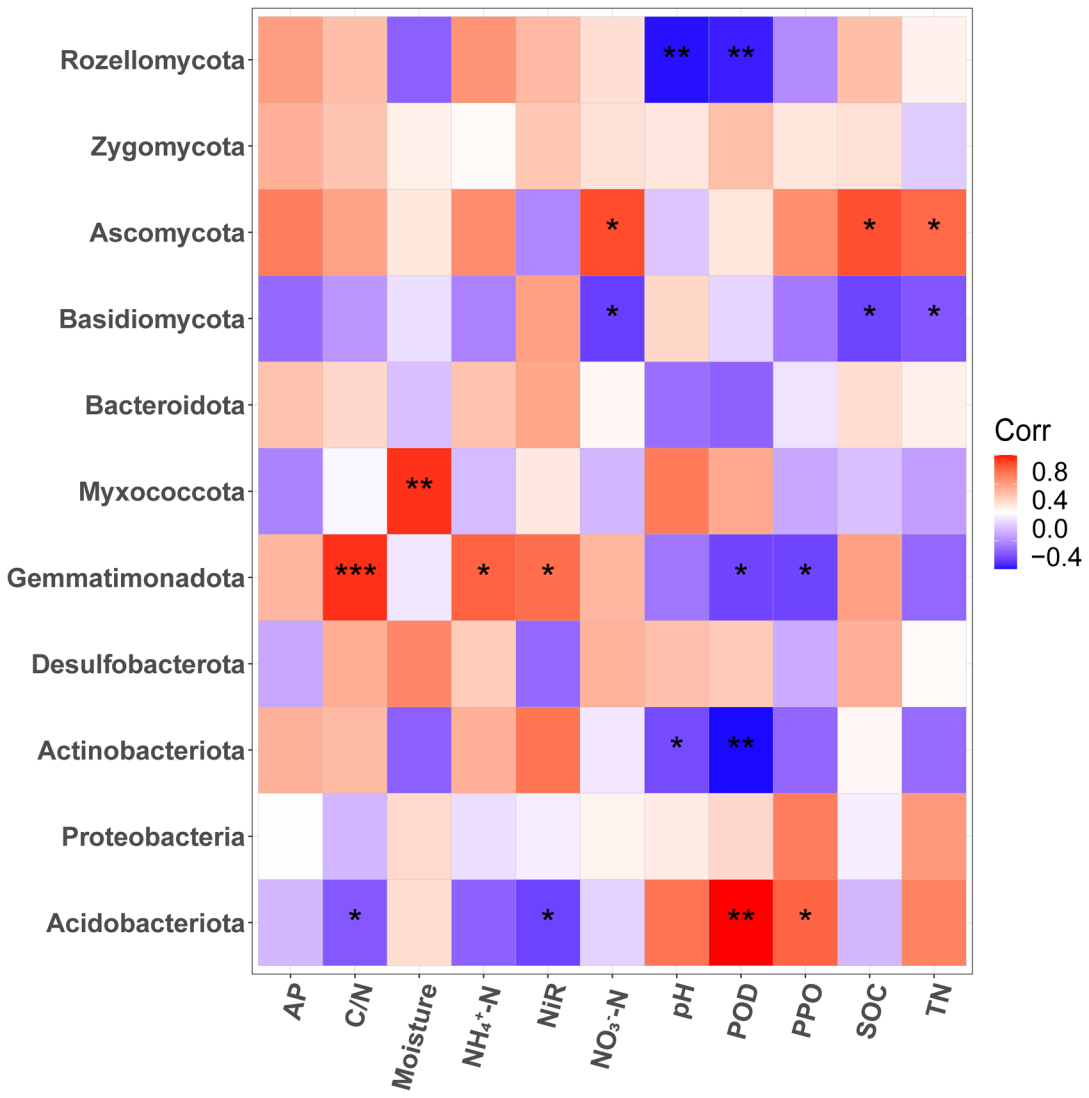
Correlation heat map between soil physicochemical properties and dominant bacterial and fungal community (phyla level) of *P. massoniana* provenances with different carbon storage levels. (* *p* < 0.05; ** *p* < 0.01; *** *p* < 0.001. AP, available phosphorus; NIR nitrite reductase; POD, peroxidase; PPO, polyphenol oxidase; SOC, Soil organic carbon; TN, total nitrogen).

The correlation heatmap (Figure 4) revealed that the phyla abundance of bacteria and fungi had no significant correlation with soil AP contents (*p* > 0.05). For fungi, Basidomycota was significantly negatively correlated with soil SOC, TN, and NO_3_^−^-N contents (*p* < 0.05). While, Ascomycota was on the opposite side which had significant negative correlation with soil SOC, TN, and NO_3_^−^-N contents (*p* < 0.05). The abundance of Rozellomycota had significant negative correlation with soil pH and the activity of POD (*p* < 0.01). For bacteria, *Acidobacteria* had significant negative correlation with C/N and NIR, positive correlation with POD and PPO (*p* < 0.05). Conversely, Gemmatimonadota had significant positive correlation with C/N, NH_4_^+^-N and NIR, negative correlation with POD and PPO (*p* < 0.05). *Myxococcota* was significantly positively correlated with moisture (*p* < 0.01). Actinobacteriota was significantly negatively correlated with pH (*p* < 0.05) and POD (*p* < 0.01).

**Figure 5:**
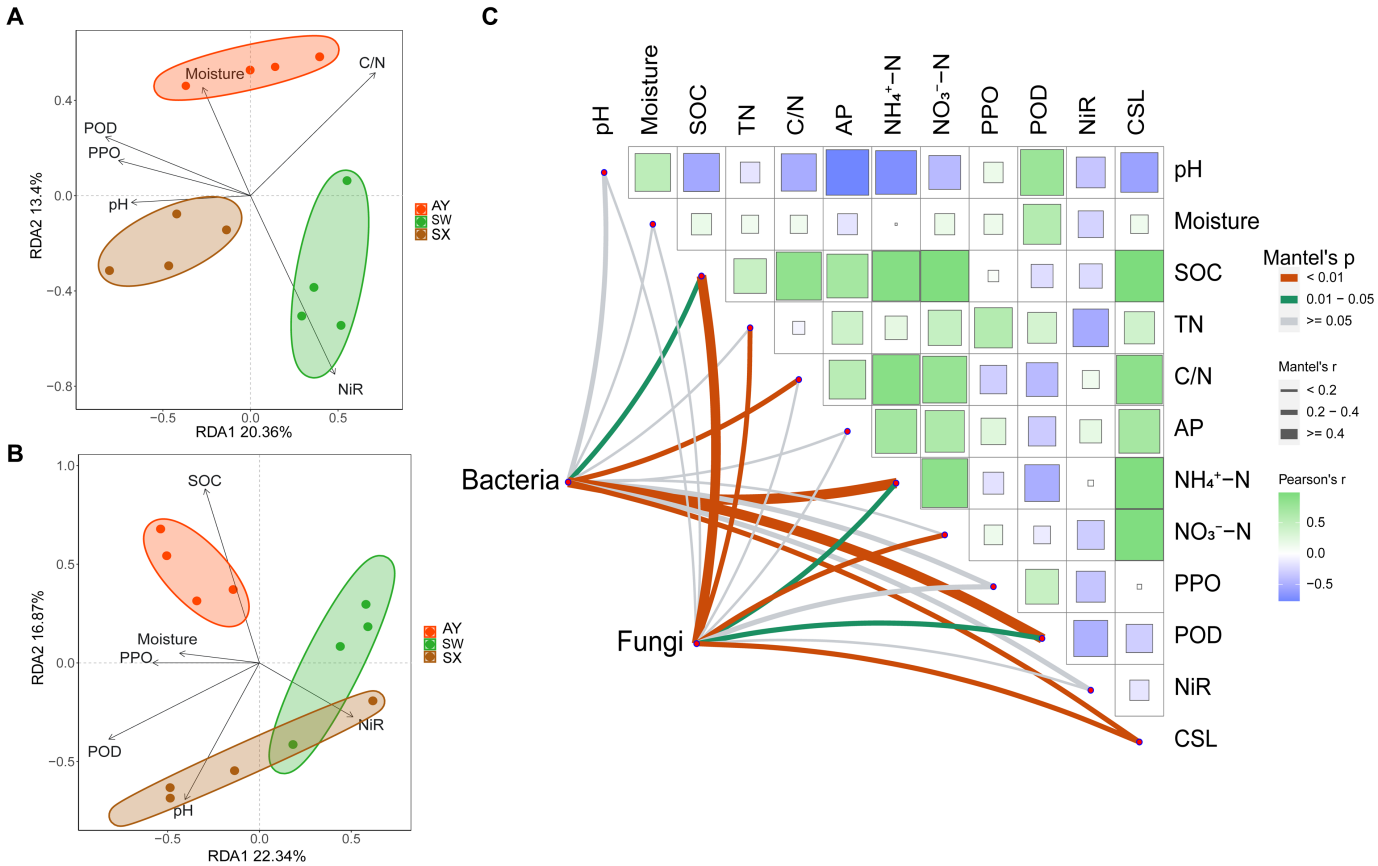
Redundancy analysis of soil bacteria **(A)** and fungi **(B)** communities and selected soil properties across three carbon storage levels in the *P. massoniana* plantation. Spearman’s correlation analysis and Mantel tests **(C)** for microbial communities with measured soil factors. (AP, available phosphorus; NIR nitrite reductase; POD, peroxidase; PPO, polyphenol oxidase; SOC, Soil organic carbon; TN, total nitrogen; CSL, carbon storage level).

The redundancy (RDA) analysis revealed the effects of soil factors on microbial community structures (Figures 5A,B). The first and second RDA axes accounted for 34.99% of the variance in the bacterial communities (Figure 5A). Soil C/N, POD, PPO and NiR activities were the main factors influencing the bacterial communities (*p* = 0.011, 0.005, 0.02, and 0.001, respectively). The Mantel test showed that SOC (r = 0.263, *p* = 0.025), C/N (*r* = 0.379, *p* = 0.008), NH_4_^+^-N (*r* = 0.432, *p* = 0.005), and POD (*r* = 0.568, *p* = 0.001) had significant correlation with effects on bacterial community structures (Figure 5C).

The first two RDA axes accounted for 42.93% of the variance in fungal communities (Figure 5B). Soil SOC content were the main factor influencing the fungal communities (p = 0.001). Furthermore, the Mantel test suggested that fungal community structures were significantly correlated with SOC (*r* = 0.425, *p* = 0.003), TN (*r* = 0.374, *p* = 0.005), NH_4_^+^-N (*r* = 0.318, *p* = 0.025), NO_3_^−^-N (*r* = 0.383, *p* = 0.007), and POD (*r* = 0.332, *p* = 0.015) (Figure 5C). In addition, the mantel test revealed that the carbon storage level of *P. massoniana* provenance significantly affected the community structure of fungi and bacteria (*p* < 0.01) (Figure 5C).

### 3.5. Putative functional prediction

**Figure 6:**
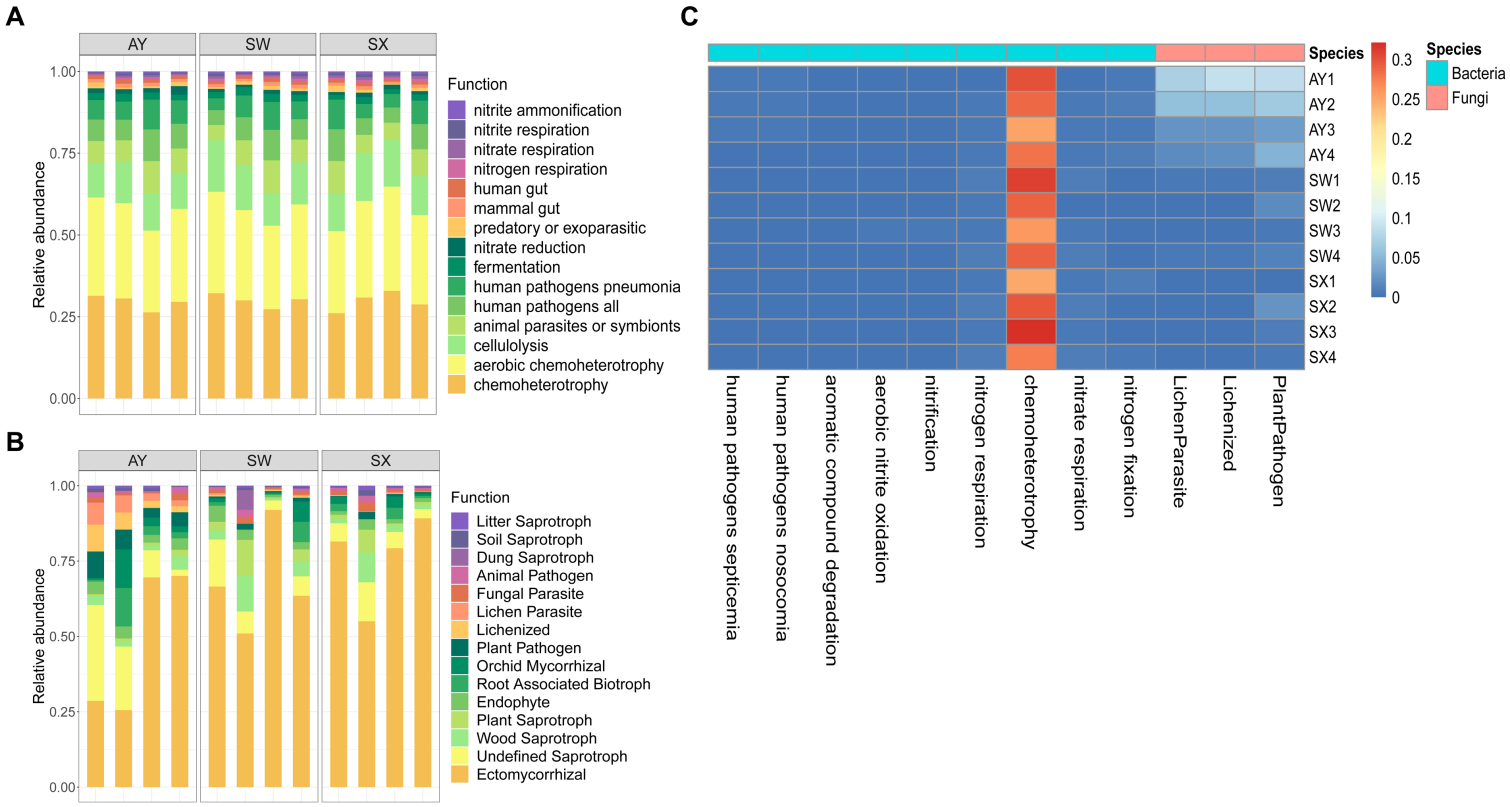
Relative abundance of bacteria **(A)** and fungal **(B)** ranking the top 15 predicted functional genes and heatmap **(C)** for functional categories with significant differences based on ANOVA analysis (*p* < 0.05). (AY, Jiangxi Anyuan provenance; SW, Fujian Shaowu provenance; SX, Zhejiang Shengxian provenance).

The bacterial function was predicted based on FAPROTAX (Figure 6A). Core functions in the bacterial community were chemoheterotrophy (29.61%), aerobic_chemoheterotrophy (28.24), cellulolysis (12.48%), animal_parasites_or_symbionts (7.47%), human_pathogens_all (7.09%), human_pathogens_pneumonia (6.43%), fermentation (2.04%), nitrate_reduction (1.29%), and predatory_or_exoparasitic (1.00%). Compared with AY and SX, SW had significantly higher aromatic_compound_degradation, aerobic_nitrite_oxidation and nitrification (*p* < 0.05). AY had significantly higher nitrogen_storage than SW and SX (*p* < 0.05), along with significantly lower nitrogen_respiration, chemoheterotrophy and nitrate_respiration. Fungal communities were divided into different trophic modes, including pathotrophs, saprotrophs, and symbiotrophs, based on the FUNGuild database (Figure 6B). Compared with SW and SX, AY provenance had significantly more lichenization, lichen parasites and plant pathogens (*p* < 0.05).

## 4. Discussion

### 4.1. Diversity, community structures, and functions of soil microorganisms at different levels of carbon storage

Our research suggested that community diversity and structures of soil microorganisms in the rhizosphere soil of *P. massoniana* provenances changed at different levels of carbon storage. Studies have shown that because the carbon metabolism cycle of fungal mycelia is shorter than that of bacteria (Staddon et al., 2003), the fungi response to the carbon storage effect of plant photosynthesis is more obvious than that of bacteria (Jones et al., 2009), which our research confirms. We did not find significant difference in bacterial alpha diversity of *P. massoniana* rhizosphere soil at different carbon storage levels. The Chao1 and Ace diversity of fungi (Figure 1) in the *P. massoniana* rhizosphere soil with a high carbon storage level was significantly higher than that with a low carbon storage level. Researchers (Huang F. et al., 2019) have reported that higher fungal diversity can ensure agroecosystem stability and sustainable crop production, which indicated that the rhizosphere ecosystem of AY *P. massoniana* provenance representing high carbon storage levels has greater stability than lower carbon storage levels. Our results indicated that under a high carbon storage level, the Chao1 and ACE values of fungi in the *P. massoniana* rhizosphere soil were significantly increased, but the Simpson index and Shannon index did not change significantly. Thus, the carbon storage level of *P. massoniana* affected the number of fungal species but not diversity.

The PCoA analysis indicated that the microbial community compositions and structures significantly changed under different levels of carbon storage of *P. massoniana* provenances. The bacteria that shifts across carbon storage levels were mainly Acidobacteriota, Proteobacteria and Actinobacteriota. Generally, there are extensive studies on Proteobacteria in soil science. Proteobacteria is a diverse bacterium preferring to grow in soil with high effective carbon levels (Fierer et al., 2007). Some Proteobacteria strains are plant growth-promoting bacteria and participate in the nitrogen storage process (Hassen et al., 2011). Actinobacteriota are low-nutrient species that prefer nutrient-poor environments (Liu et al., 2019). These bacteria usually predominate in dry, low nutrition and poor conditions (Maestre et al., 2015). Studies (Eichorst et al., 2018) have shown that Acidobacteriota uses organic nitrogen, inorganic nitrogen, as well as various carbohydrates, as the nitrogen source. It plays a vital role in organic matter decomposition as well as nutrient cycling. The results of FAPROTAX-based online functional prediction (Figure 6C) shows that the carbon storage level of *P. massoniana* provenance changed the carbon and nitrogen metabolism in the rhizosphere, increased the nitrogen storage effect, and decreased the nitrite respiration, chemical energy heterotrophic and nitrate respiration.

The fungal taxa with high relative abundance were Ascomycota and Basidiomycota in the studied soils (Zhang X. et al., 2020). The PCoA and LEfSe analysis results revealed that fungal community structures significantly shifted across different carbon storage levels, especially for Ascomycota and Basidiomycota. Basidiomycota can effectively degrade lignocellulose organics, participating in litter decomposition (Yelle et al., 2008). Therefore, we inferred that variation in Basidiomycota was directly related to the change in soil SOC content. In addition, we found that Ascomycota in the *P. massoniana* rhizosphere had more significant competitive advantages at high carbon storage provenances. Ascomycota is the key driving factor of the carbon and nitrogen cycles (Cook, 2015). Ascomycota plays an important role in *P. massoniana* rhizosphere microorganism response to carbon storage levels through its role in these cycles. Additionally, FUNGuild analysis (Figure 6C) showed that at high carbon storage levels, the lichenized fungi in the *P. massoniana* rhizosphere increased, which indicated that the nutrient pattern of fungi had changed.

### 4.2. Biomarkers in different levels of carbon storage

The LDA analysis of bacteria showed that *Acidobacteriales, Acidobacteriae, Acidobacteriae_Subgroup_1, Alphaproteobacteria* were mainly enriched in SX. Most of these taxa belong to the Acidobacteria phylum. It has been shown that Acidobacteria usually grow in soils with low nutrient levels (Pankratov and Dedysh, 2010). SX is low carbon storage provenance with low content of organic carbon, ammonium nitrogen, nitrate nitrogen in the rhizosphere. It can enrich by acidophilus phylum to decompose cellulose and other carbon compounds to obtain organic carbon and other nutrients required for growth. The biomarkers of SW are *Thermoleophilia, Gaiellales, Elsterales,* and *Gemmatimonadaceae*. Compared to the SX group, SW representing medium carbon storage had higher effective nitrogen content, which may have led to the enrichment of *Thermoleophilia* and *Gaiellales* (Ramirez, 2011). Besides, *Gemmatimonadaceae* is plant-promoting bacteria. It protect plant health and promote plant growth by secreting antibiotics that inhibit or kill microorganisms that are harmful to plants, thus indirectly leading to an increase in plant carbon storage (Hui et al., 2020).

The LDA analysis of fungi showed that the biomarkers of SX were *Boletaceae* and *Pulveroboletus,* which were suitable for growth between pH of 5–6 (Wu et al., 2014). The pH of SX was 5.05, slightly higher than that of SW and AY. Our hypothesis is that the increase in pH caused by SX may have led to an enrichment of *Boletaceae* and *Pulveroboletus*. SW is enriched with more *Rhizopogonaceae*. This results suggested that medium carbon storage provenance obtained more nitrogen nutrients by altering the biological strategy of the fungi compared to low carbon storage provenance (Zeng et al., 2021). In addition, more diverse fungal taxa were enriched in AY provenance, including *Archaeorhizomycetales, Sebacinaceae, Archaeorhizomycetales, Atheliaceae, Tremellodendron,* and *Thelephora. Archaeorhizomyces* has been demonstrated to have the ability to utilize either glucose or cellulose as a carbon source for growth (Menkis et al., 2014). The rhizosphere soil of high carbon storage provenance exhibits elevated levels of organic carbon content, which is influenced by apoplastic material and root secretion. This may result in an increased proportion of decomposers. The presence of *Atheliaceae, Sebacinaceae, Tremellodendron,* and *Thelephora* ectomycorrhizal fungi suggested that high carbon storage provenance exhibit greater diversity in nutrient acquisition strategies (Weiss et al., 2004; Smith et al., 2009).

### 4.3. Factors driving the dissimilarity of microbial community

In our research, RDA and Mantel tests revealed that the NH_4_^+^-N, PER contents and C/N had the most significant effect (*p* < 0.01) on bacterial community structures. A potential reason why C:N ratio can significantly affect bacterial communities may be that microorganisms disrupt elemental stoichiometric balance and homeostasis (Bi et al., 2021). Therefore, the C:N ratio is an important marker of changes in microbial communities. We found that NH_4_^+^-N had a significant correlation with the bacterial communities in the rhizosphere soil, which indicated that variables associated with nitrogen transformations may play an important role in the changes of bacterial community structure (Zhang et al., 2021a). Some studies believe that for subtropical forest ecosystems with generally acidic soil, compared to NO_3_^−^-N, NH_4_^+^-N has a more important effect on the bulk soil and soil aggregates, which further affects the microbial community structure (Yan et al., 2017). Studies has shown that increasing the content of NH_4_^+^-N will enable more bacterial groups to participate in the recycling of carbon and nitrogen. In addition to the correlation with humus formation (Yan et al., 2017), the POD enzyme also had a significant positive correlation with *Actinobaciota, Gemmatimonadota and Rozellomycota*. Besieds, RDA and Mantel tests showed that the SOC, TN, and NO_3_^−^-N contents had the most significant effect (*p* < 0.01) on the fungal community structure. As the energy source of microorganisms, the increase in SOC content will significantly affect the microbial metabolic cycle (Sokol and Bradford, 2019). Research has proven that SOC is the direct influencing factor regulating microbial community structure (He et al., 2014). The significant effect of TN on the fungal community structure indicated that TN may limit the carbon utilization by microorganisms (Allen and Schlesinger, 2004). In this study, the nitrate decomposition rate decreased with the increase in *P. massoniana* carbon storage levels, which indicates that plant carbon storage levels may change the nitrification rate of rhizosphere soil. The significant impact of NO_3_^−^-N on the fungal community structure indicated that nitrogen conversion rates of rhizosphere soil played an important role in shaping microbial communities (Wang et al., 2022). Generally, variations in carbon storage levels among *P. massoniana* provenances lead to differences in the carbon cycle of rhizosphere microorganisms in the *P. massoniana* forest ecosystem, which also leads to the nutrient preference of soil microorganisms.

Most studies have shown that the AP content in soil is an important driving factor affecting soil microbial diversity, which can improve soil microbial diversity (Tan et al., 2013; Siciliano et al., 2014). However, in our study, soil AP content had no effect on microbial diversity and community structures. Other researchers (Liu and Jiang, 2018) also obtained similar experimental conclusions. This may be related to the small range of AP changes in this study. It has been reported (Jian et al., 2021) that the overall soil comprehensive fertility of *P. massoniana* forests is poor, and the AP content is low. Long-term low phosphorus stress causes specific microbial communities to be screened out and adapt to this growth condition for a long time (Ky et al., 2020). Microorganisms in low phosphorus-stressed environments were less affected by this than in soil environments with higher AP levels. Therefore, AP was not a driver of microbial community change in the *P. massoniana* forest ecosystem.

### 4.4. Ecological significance of microbial changes in the *Pinus massoniana* provenances rhizosphere

This study confirmed that microbial diversity and community structure were significantly different in the rhizosphere of *P. massoniana* provenances under different carbon storage levels. For *P. massoniana* provenances, in the case of small differences in environmental factors, a very important reason for the variation of carbon storage among provenances is due to their own factors, which are mainly reflected in the wood properties of the standing trees and the strength of carbon storage related metabolic pathways (Hou et al., 2016). It has been shown (Yin et al., 2010) that newly fixed plant carbon is allocated to the lower part of the ground for root growth and is continuously lost in the form of rhizosphere sediment. Differences in the amount of carbon storage affect to some extent the type and metabolic rate of plant root metabolites which contains high molecular aromatic compounds and low molecular organic acids, resulting in altered soil sediments (Bi et al., 2021). This will further affect the soil physicochemical properties and microorganisms. Microorganisms are also influenced by the organic matter decomposition, apoplast, nutrient requirements and root turnover (Bi et al., 2022). Variations in organic matter and nutrient content can stimulate microorganisms to secrete more extracellular enzymes that accelerate soil nutrient activation (Luan et al., 2022). In turn, these enzymes can promote nutrient uptake by plant roots. This process can illustrate the relationship between plant–microbe interactions more graphically. A number of researchers have been working on carbon storage in forests from different fields and perspectives (Geddes et al., 2014). From the perspective of plant–microbial interaction, we selected *P. massoniana* provenances that establish better interaction with environmental microorganisms. While providing excellent varieties for the construction of high carbon storage artificial forests of *P. massoniana*, we reasonably analyzed the relationship between the carbon storage level of *P. massoniana* provenances and the environment, which has certain guiding significance for the scientific management of carbon storage forestry in the future. In this study, we believe that AY provenance representing high carbon storage levels have more abundant rhizosphere microbial diversity, which represents a more stable ecological structure of the rhizosphere microenvironment. Therefore, AY provenance is more suitable for building high carbon storage plantations for the purpose of sustainability. In contrast to a series of previous studies (Chen et al., 2022; Zhou et al., 2022) on *P. massoniana* seedlings, the *P. massoniana* plantation studied in this paper is an overmature forest. The response of rhizosphere microorganisms to carbon storage capacity and environmental factors at different growth stages is provided with more diversified reference significance and value. Under the new dynamics of carbon neutrality targets, future studies on the interactions between carbon storage and the effects of *P. massoniana* rhizosphere microenvironments under the coupling of multiple environmental factors (light, water, CO_2_ concentration) should also be reinforced.

## 5. Conclusion

The results revealed that there were significant differences in the microbial community structure of the *P. massoniana* interrhizosphere soil at different carbon storage levels. Soil fungi responded more significantly than bacteria to alterations in carbon storage in *P. massoniana*. The bacterial diversity did not change. While, the fungal diversity of *P. massoniana* at high carbon storage levels was higher. Compared with other provenances, AY provenance, which represent high carbon storage level, are more suitable for the construction of high carbon storage forest ecosystems for the purpose of sustainability. C/N, NO_3_^−^-N, NH_4_^+^-N, and SOC were the main drivers shaping the interrooted bacterial and fungal communities. The effect of PER enzyme activity confirmed that changes in soil microbial communities are highly correlated with carbon cycling. AP content had no significant effect on the formation of *P. massoniana* interrooted microbial communities. Generally, changes in the level of carbon storage in *P. massoniana* provenance led to variations in the rhizosphere soil carbon cycle, as well as variations in the nutrient preferences of soil microorganisms, which resulted in differences in the rhizosphere microbial community structure.

## Data availability statement

The original contributions presented in the study are included in the article/Supplementary material, further inquiries can be directed to the corresponding author.

## Author contributions

ZH: Investigation, Software, Writing – original draft. XH: Investigation, Methodology, Writing – review & editing. CZ: Software, Supervision, Writing – review & editing. MZ: Conceptualization, Writing – review & editing. JW: Writing – review & editing. YH: Conceptualization, Software, Writing – review & editing. DW: Methodology, Software, Writing – review & editing. SY: Conceptualization, Writing – review & editing. QY: Writing – review & editing. KJ: Writing – review & editing.

## Footnotes

^1^http://www.zoology.ubc.ca/louca/FAPROTAX/

^2^https://github.com/UMNFuN/FUNGuild
