# Supplementary material for: Microbial communities and functions changed in rhizosphere soil of Pinus massoniana provenances with different carbon storage
Source: Front Microbiol. 2023 Nov 3;14:1264670. doi: 10.3389/fmicb.2023.1264670 (PMC10655096; doi:10.3389/fmicb.2023.1264670)
Supplement: Supplementary file 1 [file Data_Sheet_1.docx]

**Supplementary Materials**

Table of Contents:

**Figure. S1** District distribution map of sampling site.

**Figure. S2** ANOSIM Analysis (analysis of similarities) in bacteria and fungi of soil of *P. massoniana* provenance at different carbon sequestration levels based on bray-curtis distance.

**Table S1** Relative abundances of the main soil microbial phyla of *P. massoniana* provenance at different carbon sequestration levels.

**Table S2** Differences identified using LefSe in bacteria and fungi of soil of *P. massoniana* provenance at different carbon sequestration levels (LDA score>3).


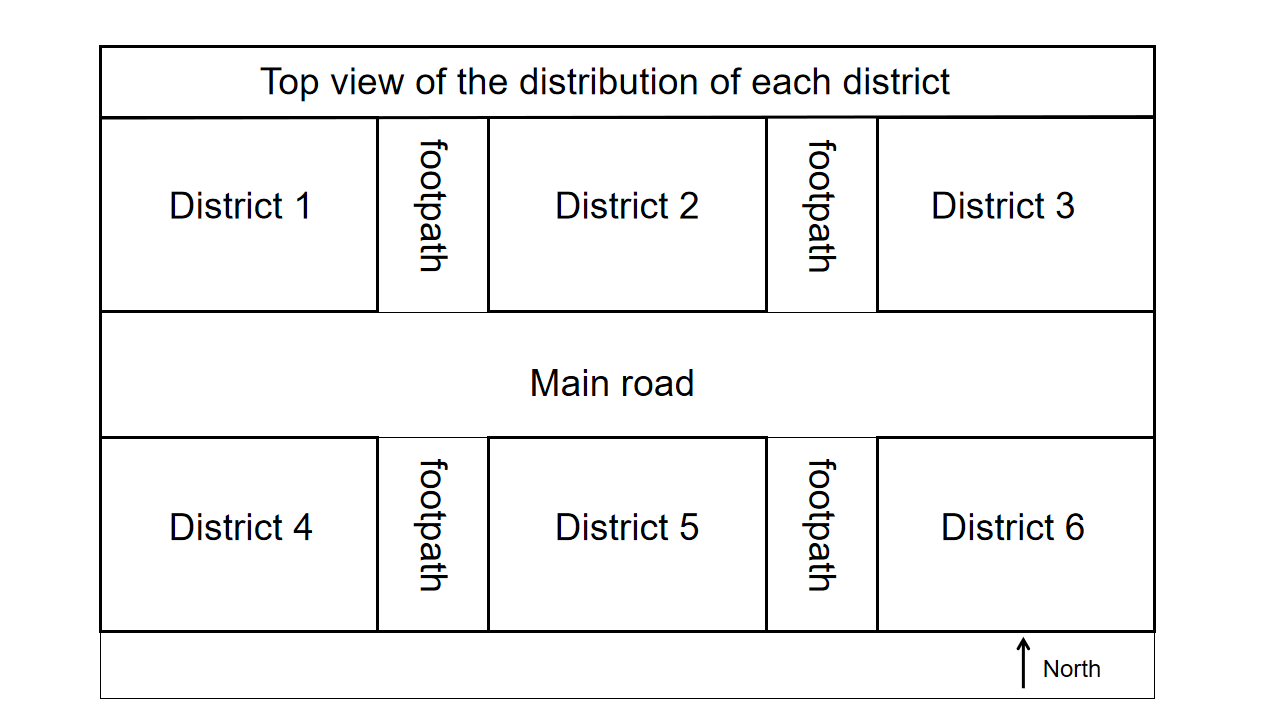


Figire.S1 District distribution map of sampling site.


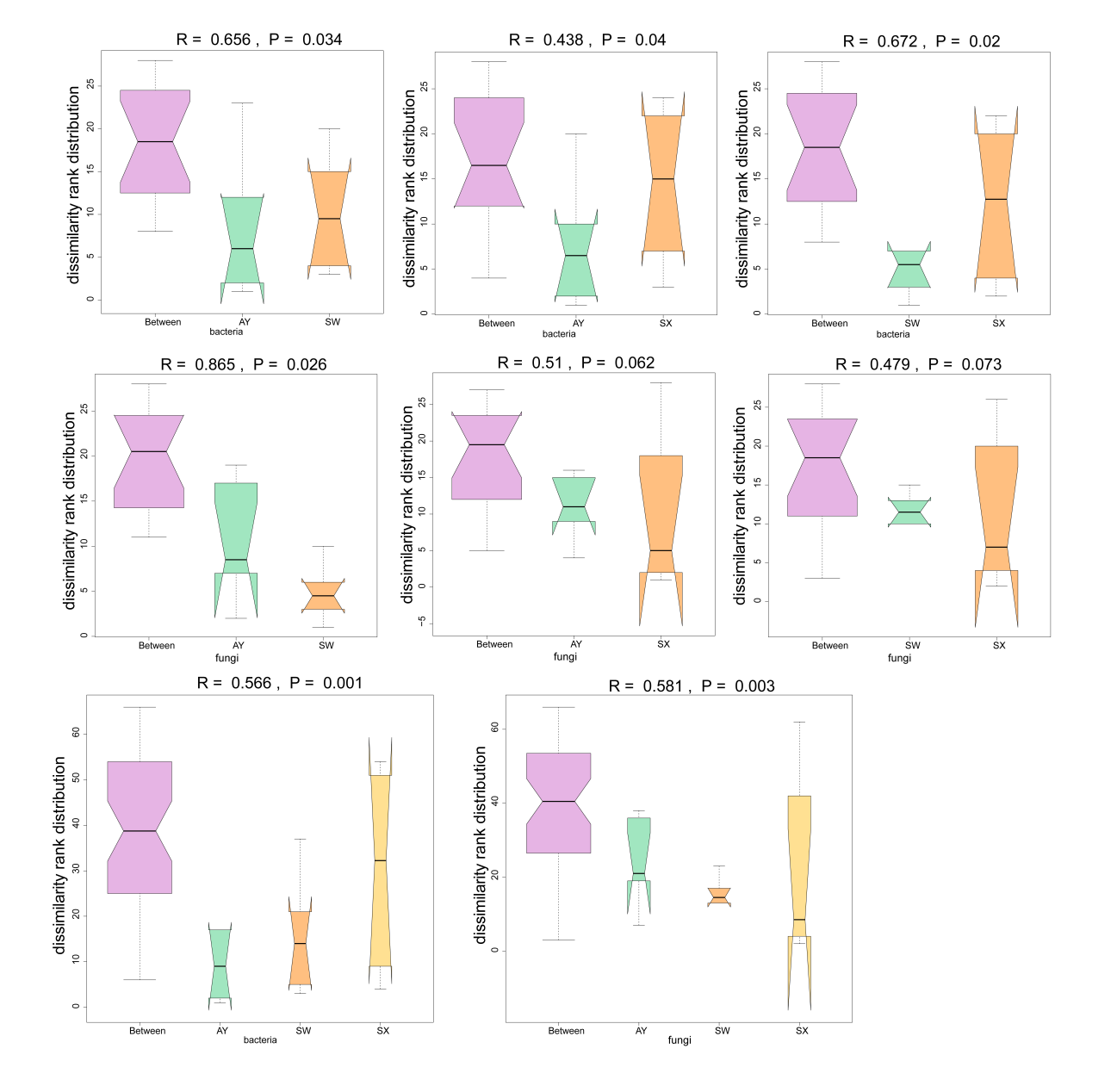


Figure.S2 ANOSIM Analysis (analysis of similarities) in bacteria (a) and fungi (b) of soil of *P. massoniana* provenance at different carbon sequestration levels based on bray-curtis distance. (Note: Between, [differences between groups](javascript:;); AY, differences within Jiangxi Anyuan provenance; SW, differences within Fujian Shaowu provenance; NZ, differences within Shanxi Nanzhen provenance).

TableS1 Relative abundances of the main soil microbial phyla of *P. massoniana* provenance at different carbon sequestration levels.

|  | AY (%) | SW (%) | SX (%) |
| --- | --- | --- | --- |
| Bacteria |  |  |  |
| Acidobacteriota | 44.79 ± 0.73b | 41.12 ± 0.61c | 46.90 ± 0.55a |
| Proteobacteria | 32.74 ± 1.34 | 32.23 ± 0.48 | 32.73 ± 0.81 |
| Actinobacteriota | 12.32 ± 0.91b | 16.07 ± 0.34a | 11.90 ± 1.36b |
| Desulfobacterota | 2.34 ± 0.40 | 1.32 ± 0.50 | 1.71 ± 0.43 |
| Gemmatimonadota | 1.69 ± 0.30a | 2.62 ± 0.43a | 0.65 ± 0.18b |
| Myxococcota | 1.40 ± 0.11 | 1.58 ± 0.38 | 1.66 ± 0.23 |
| Bacteroidota | 1.39 ± 0.14 | 1.59 ± 0.07 | 1.30 ± 0.09 |
| RCP2-54 | 0.82 ± 0.05 | 0.76 ± 0.03 | 0.69 ± 0.11 |
| Verrucomicrobiota | 0.62 ± 0.07 | 0.48 ± 0.06 | 0.64 ± 0.06 |
| Firmicutes | 0.36 ± 0.02b | 0.54 ± 0.05a | 0.48 ± 0.04a |
| Fungi |  |  |  |
| Basidiomycota | 61.40 ± 6.52b | 79.82 ± 4.95a | 78.86 ± 2.61a |
| Ascomycota | 36.75 ± 6.13a | 18.42 ± 4.8b | 18.76 ± 2.59b |
| unidentified | 0.99 ± 0.22 | 1.03 ± 0.30 | 1.53 ± 0.58 |
| Zygomycota | 0.67 ± 0.24 | 0.61 ± 0.31 | 0.59 ± 0.32 |
| Rozellomycota | 0.06 ± 0.03 | 0.10 ± 0.03 | 0.02 ± 0.01 |
| Other | 0.01 ± 0.01 | 0.01 ± 0.00 | 0.16 ± 0.14 |
| Chytridiomycota | 0.05 ± 0.02 | 0.02 ± 0.01 | 0.05 ± 0.03 |
| Glomeromycota | 0.06 ± 0.02 | 0.01 ± 0.01 | 0.04 ± 0.02 |

Note: All data are presented as the mean ± standard error. Lowercase letters indicate that the mean relative abundances of the main soil microbial phyla are significantly different (P < 0.05) at different carbon sequestration levels.

TableS2 Differences identified using LefSe in bacteria and fungi of soil of *P. massoniana* provenance at different carbon sequestration levels (LDA score>3).

| Biomarker | Logarithm value | Groups | LDA score | p-value |
| --- | --- | --- | --- | --- |
| Bacteria |  |  |  |  |
| g_Ellin6067 | 4.23 | AY | 3.73 | 0.03 |
| o_Xanthomonadales | 4.07 | AY | 3.51 | 0.05 |
| f_Subgroup_13 | 2.84 | AY | 3.10 | 0.05 |
| f_Comamonadaceae.Other | 3.59 | AY | 3.05 | 0.02 |
| g_Klebsiella | 3.16 | AY | 3.01 | 0.01 |
| f_Rhodocyclaceae | 3.41 | AY | 3.00 | 0.03 |
| p_Actinobacteriota | 5.21 | SW | 4.36 | 0.04 |
| c_Thermoleophilia | 4.83 | SW | 4.17 | 0.03 |
| o_Gaiellales | 4.81 | SW | 4.15 | 0.03 |
| o_Gaiellales.f_.g_ | 4.77 | SW | 4.12 | 0.03 |
| o_Gaiellales.f_ | 4.77 | SW | 4.10 | 0.03 |
| o_Elsterales.f_ | 4.90 | SW | 4.07 | 0.02 |
| o_Elsterales.f_.g_ | 4.90 | SW | 4.04 | 0.02 |
| f_Gemmatimonadaceae.g_ | 4.39 | SW | 4.01 | 0.03 |
| p__Gemmatimonadota | 4.42 | SW | 4.00 | 0.03 |
| o_Gemmatimonadales | 4.42 | SW | 4.00 | 0.03 |
| f_Gemmatimonadaceae | 4.42 | SW | 3.99 | 0.03 |
| c_Gemmatimonadetes | 4.42 | SW | 3.99 | 0.03 |
| c_Acidimicrobiia | 4.33 | SW | 3.76 | 0.02 |
| o_IMCC26256 | 4.10 | SW | 3.54 | 0.01 |
| g_IMCC26256 | 4.10 | SW | 3.52 | 0.01 |
| f_IMCC26256 | 4.10 | SW | 3.52 | 0.01 |
| g_Gaiella | 3.70 | SW | 3.35 | 0.02 |
| f_Gaiellaceae | 3.70 | SW | 3.35 | 0.02 |
| o_Bacteroidales | 3.79 | SW | 3.27 | 0.02 |
| o_Acidimicrobiales | 3.54 | SW | 3.14 | 0.05 |
| f_Acidimicrobiaceae.g_ | 3.54 | SW | 3.14 | 0.05 |
| f_Acidimicrobiaceae | 3.54 | SW | 3.11 | 0.05 |
| g_Acinetobacter | 2.36 | SW | 3.05 | 0.03 |
| g_MND1 | 3.32 | SW | 3.04 | 0.04 |
| o_Acidobacteriales | 5.34 | SX | 4.54 | 0.01 |
| p_Acidobacteriota | 5.67 | SX | 4.42 | 0.01 |
| c_Acidobacteriae | 5.67 | SX | 4.28 | 0.02 |
| c_Alphaproteobacteria | 5.35 | SX | 4.16 | 0.04 |
| f_Acidobacteriaceae_Subgroup_1_ | 4.87 | SX | 4.15 | 0.03 |
| o_Rhizobiales | 4.89 | SX | 3.84 | 0.02 |
| f_Acidobacteriaceae_Subgroup_1_.g_ | 4.46 | SX | 3.73 | 0.03 |
| o_Acetobacterales | 3.66 | SX | 3.16 | 0.01 |
| f_Acetobacteraceae | 3.66 | SX | 3.11 | 0.01 |
| g_Luedemannella | 3.43 | SX | 3.07 | 0.04 |
| Fungi |  |  |  |  |
| o_Archaeorhizomycetales | 5.02 | AY | 4.63 | 0.02 |
| c_Archaeorhizomycetes | 5.02 | AY | 4.63 | 0.02 |
| f_Sebacinaceae | 4.95 | AY | 4.61 | 0.04 |
| o_Sebacinales | 4.95 | AY | 4.60 | 0.04 |
| o_Archaeorhizomycetales.f_.g_ | 4.91 | AY | 4.59 | 0.03 |
| o_Archaeorhizomycetales.f_ | 4.91 | AY | 4.56 | 0.03 |
| f_Atheliaceae | 4.55 | AY | 4.31 | 0.01 |
| o_Atheliales | 4.55 | AY | 4.29 | 0.01 |
| f_Atheliaceae.g_ | 4.55 | AY | 4.28 | 0.01 |
| g_Tremellodendron | 4.43 | AY | 4.18 | 0.01 |
| g_Thelephora | 1.56 | AY | 4.08 | 0.03 |
| g_Cordyceps | 2.55 | AY | 3.81 | 0.04 |
| o_Pezizales.f_ | 2.97 | AY | 3.65 | 0.01 |
| o_Pezizales.f_.g_ | 2.97 | AY | 3.64 | 0.01 |
| f_Capnodiales_fam_Incertae_sedis | 3.70 | AY | 3.45 | 0.01 |
| g_Capnobotryella | 3.70 | AY | 3.43 | 0.01 |
| f_Agaricaceae | 3.67 | AY | 3.42 | 0.03 |
| f_Agaricaceae.g_ | 3.67 | AY | 3.41 | 0.03 |
| o_Capnodiales | 3.71 | AY | 3.41 | 0.01 |
| c_Lecanoromycetes | 3.48 | AY | 3.40 | 0.04 |
| p_Basidiomycota.c_.o_.f_.g_ | 3.60 | AY | 3.36 | 0.02 |
| p_Basidiomycota.c_.o_.f_ | 3.60 | AY | 3.36 | 0.02 |
| .p_Basidiomycota.c_.o_ | 3.60 | AY | 3.35 | 0.02 |
| p_Basidiomycota.c_ | 3.60 | AY | 3.34 | 0.02 |
| o_Boletales | 5.60 | SW | 5.24 | 0.02 |
| f_Rhizopogonaceae | 5.55 | SW | 5.21 | 0.04 |
| g_Rhizopogon | 5.51 | SW | 5.15 | 0.05 |
| o_Helotiales.f_ | 4.18 | SW | 3.81 | 0.03 |
| f_Vibrisseaceae | 4.20 | SW | 3.81 | 0.02 |
| g_Phialocephala | 4.20 | SW | 3.79 | 0.02 |
| o_Helotiales.f_.g_ | 4.18 | SW | 3.75 | 0.03 |
| f_Boletaceae | 4.93 | SX | 4.63 | 0.04 |
| g_Pulveroboletus | 4.91 | SX | 4.60 | 0.03 |
| g_Wilcoxina | 4.09 | SX | 3.76 | 0.04 |
| f_Pyronemataceae | 4.09 | SX | 3.76 | 0.02 |
| f_Geminibasidiaceae | 4.05 | SX | 3.71 | 0.02 |
| g_Geminibasidium | 4.05 | SX | 3.70 | 0.02 |
| c_Wallemiomycetes | 4.05 | SX | 3.69 | 0.02 |
| o_Geminibasidiales | 4.05 | SX | 3.69 | 0.02 |
| c_Orbiliomycetes | 3.72 | SX | 3.38 | 0.04 |
| f_Orbiliaceae | 3.69 | SX | 3.34 | 0.04 |
| o_Orbiliales | 3.69 | SX | 3.34 | 0.04 |
| g_Hyalorbilia | 3.25 | SX | 3.17 | 0.03 |
